# Clinical characteristics, disease course, and clinical subgroups of young adults with type 2 diabetes

**DOI:** 10.1210/jendso/bvag100

**Published:** 2026-04-24

**Authors:** Anu Sharma, Jose M Lazaro-Guevara, Devorah Stucki, Sanjiv Anand, Corrine K Welt, Marcus G Pezzolesi

**Affiliations:** Division of Endocrinology, Metabolism and Diabetes, University of Utah School of Medicine, Salt Lake City, UT 84108, USA; Division of Endocrinology, Diabetes and Metabolism, University of Florida College of Medicine, Gainesville, FL 32610, USA; Department of Medical Genetics, McGill University Health Center (MUHC), Montreal, QC H3H 1P3, Canada; Division of Experimental Medicine, Faculty of Medicine, McGill University, Montreal, QC H4A 3J1, Canada; Department of Internal Medicine, Division of Nephrology and Hypertension, University of Utah School of Medicine, Salt Lake City, UT 84112, USA; Department of Internal Medicine, Division of Nephrology and Hypertension, University of Utah School of Medicine, Salt Lake City, UT 84112, USA; Intermountain Medical Center, Transplant Services, Murray, UT 84107, USA; Division of Endocrinology, Metabolism and Diabetes, University of Utah School of Medicine, Salt Lake City, UT 84108, USA; Department of Internal Medicine, Division of Nephrology and Hypertension, University of Utah School of Medicine, Salt Lake City, UT 84112, USA; Department of Human Genetics, University of Utah School of Medicine, Salt Lake City, UT 84112, USA

**Keywords:** type 2 diabetes, young adults, subgroups, phenotype, risk factors, complications, disease progression

## Abstract

**Context:**

Type 2 diabetes (T2D) in young adults is an emerging entity distinct from that in older adults.

**Objective:**

To identify unique clinical subgroups among young adults using clinical variables.

**Methods:**

Utilizing the Utah Diabetes Database, a retrospective cohort study was performed with data from 56 327 individuals with T2D as defined by International Classification of Diseases codes and analyzed by predefined groups: young adults (18-44 years) and older adults (≥45 years). De novo k-means clustering was performed.

**Results:**

Young adults with T2D (yT2D) were more likely to be female (51%), self-identify as racial/ethnic minorities (24%), and have obesity (68%). Compared to older adults, yT2D at baseline had worse glycemic control (hemoglobin A1c [HbA1c] 7.8 ± 2.5% vs 7.3 ± 2.0%, *P* < .001), higher body mass index (BMI; 34 ± 9 vs 32 ± 7 kg/m^2^, *P* < .001) and higher estimated glomerular filtration rate (eGFR; 100 ± 25 vs 73 ± 23 mL/min/1.73 m^2^, *P* < .001). Over time, the increase in HbA1c and BMI was greater in yT2D compared with older adults (*P* < .01) with no difference in eGFR change. Three unique clusters were determined: cluster 1 had higher BMI and lower HbA1c; cluster 2 had a higher HbA1c and was more likely to need insulin; and cluster 3 had a lower eGFR and more diabetes-related comorbidities.

**Conclusion:**

yT2D have worse glycemic control, greater obesity, and similar renal decline compared with older adults with T2D. A high-risk subgroup of yT2D presents with a significantly lower eGFR. More research is needed to determine whether targeted therapy to high-risk groups will reduce diabetes-related complications.

Type 2 diabetes (T2D) in young adults is increasing in prevalence worldwide, adding to the global burden of T2D and the associated cardiometabolic complications. T2D in young adults is also referred to as young-onset or early-onset T2D. The age defining a young adult has varied [1-4]; however, it is well established that the earlier the age at which T2D is first diagnosed, the higher the overall burden of diabetes-related morbidity and overall mortality [3-7]. In addition, the progression to β-cell failure is more rapid when T2D begins in adolescence or young adulthood, suggesting a more aggressive disease process [8, 9]. Most data stem from T2D in children and adolescents ages 10 to 17 years and have been extrapolated to young adults.

Population-level data from the United Kingdom and Hong Kong [2, 10] have highlighted the increasing burden of T2D in young adults. Several factors influence both the development and the clinical course of T2D in young adults. Some of these risk factors include socioeconomic factors, underlying genetic risk, obesity, and overall glycemic control. However, these factors do not predict the development of diabetes-related complications. A 5-subgroup novel classification of T2D was described in 2018 and was associated with diabetes-related outcomes [11]. These subgroups have been replicated in many countries to determine risk among young adults ages 18 to 45 years, with success in identifying severe insulin deficiency and mild obesity–related subgroups [1]. However, the racial and ethnic heterogeneity in the United States population differs from that previously studied. In addition, the currently recommended standard clinical monitoring for a routine clinical diabetes visit in the United States [12] presents a significant barrier to translating these subgroups into clinical care. This is because fasting insulin and C-peptide concentrations are not routinely measured in the clinical care of a patient with T2D. These values are required to calculate the homeostasis model assessment to estimate insulin resistance (HOMA2-IR) and β-cell function (HOMA2-B)—2 of the 6 variables used to define the 5 novel subgroups. In addition, the insulin assay varies by the platform used and is not standardized across clinical laboratories, which prohibits recommending these calculations across populations [13].

We therefore aimed to describe the characteristics of young adults with T2D (18-44 years) and their clinical course, including diabetes-related comorbidities. We also sought to perform clinically applicable subgrouping to predict outcomes utilizing our large population database, which includes individualized electronic health record data with routine clinical variables. We then compared the clinical characteristics at diagnosis and progression of disease during follow-up between young and older adults with T2D. Finally, we performed subgrouping in young adults with T2D.

## Materials and methods

### Study population

Approval with a waiver of consent and authorization was obtained from the institutional review boards of the University of Utah and Intermountain Health Care and the Utah Resource for Genetic and Epidemiological Research prior to the start of study activities. Utilizing the Utah Diabetes Database (UDDb) [14], individuals with T2D were identified by International Classification of Diseases, Ninth and Tenth Revision, codes. If International Classification of Diseases codes were not consistent for the type of diabetes, the type of diabetes assigned is the diagnosis where the percentage of diabetes codes associated with the diagnosis is ≥66%. The UDDb is a collaborative database between the University of Utah and Intermountain Health Care with longitudinal health data for >370 000 individuals with both type 1 diabetes and T2D [14]. These 2 health systems (University of Utah and Intermountain Health Care) serve 85% of the population in Utah. Of the 352 826 adults with T2D identified, 56 327 were included in the final analysis (Fig. S1) [15]. Individuals were excluded due to missing data at baseline (eg, no body mass index [BMI] or hemoglobin A1c [HbA1c] at diagnosis) or with values outside the clinical range (eg, BMI < 15 kg/m^2^, BMI > 70 kg/m^2^, HbA1c < 4% or 20 mmol/mol, HbA1c > 18% or 173 mmol/mol, glomerular filtration rate [GFR] > 120 mL/min/1.73 m^2^) or ≥5 SDs from the mean, laboratory values consistent with type 1 diabetes (positive glutamic acid decarboxylase 65 antibodies – specific assay manufacturer and catalog information were not available due to the retrospective nature of the study) or insulin deficiency (C-peptide concentration < 1.0 ng/mL or <0.3 nmol/L).

Baseline and follow-up clinical and laboratory data were retrieved for all included individuals. The age at diagnosis was defined by the date of first entry into the UDDb or the first date a diagnosis code for T2D was associated with the person. Young adults were defined as individuals ages 18 to 44 years at the first diagnosis of T2D, and older adults as those ages ≥45 years. The duration of follow-up was calculated as the time from an individual's first HbA1c to the last HbA1c in the database. Hypertension was defined by diagnosis code and/or a prescription for an antihypertensive agent (renin-angiotensin system blocker, diuretic, beta blocker, calcium channel blocker, or vasodilators). Nephropathy was defined by diagnosis code and/or an estimated GFR (eGFR) of <90 mL/min/1.73 m^2^) or urine albumin to creatine ratio ≥30 mg/g. Other comorbidities, including neuropathy, retinopathy, diabetic ketoacidosis (DKA), hyperglycemic hyperosmolar state (HHS), hypoglycemia, peripheral vascular disease (PVD), and foot ulcer, were defined by diagnosis codes. Only outpatient medications prescribed and outpatient laboratory data (HbA1c, creatinine, eGFR, aspartate aminotransferase [AST], alanine aminotransferase, platelets, and urine albumin) were collected and analyzed. AST-to-platelet ratio index (APRI) and fibrosis-4 index (FIB-4) were calculated at baseline and at follow-up when available.

eGFR was calculated using the Chronic Kidney Disease Epidemiology Collaboration creatinine formula [16]. APRI was calculated as


$$\begin{array}{rl} \mathrm{APRI} & =[\left(\mathrm{AST}\;\mathrm{in}\;\mathrm{IU}/L)/(\mathrm{AST}\;\mathrm{upper}\;\mathrm{limit}\;\mathrm{of}\;\mathrm{normal}\;\mathrm{in}\;\mathrm{IU}/L\right)]\\ & \;÷(\mathrm{Platelets}\;\mathrm{in}\;10^{9}/L) \end{array}$$

where the upper limit of normal for AST was 35 IU/L, and an APRI >0.5 [17] was defined as indicative of being at risk for hepatic fibrosis from metabolic dysfunction-associated steatotic liver disease (MASLD).

FIB-4 was calculated for those ages 35 to 65 years as


$$\begin{array}{rl} \mathrm{FIB}\text{-}4\; & =[(\mathrm{Age}\times (\mathrm{AST}\;\mathrm{in}\;\mathrm{IU}/L)]÷[(\mathrm{Platelets}\;\mathrm{in}\;10^{9}/L)\\ & \;\times \sqrt{\left(\mathrm{ALTinIU}/L)\right]} \end{array}$$

where FIB-4 ≥ 1.3 was defined as indicative of being at risk for hepatic fibrosis from MASLD.

### Statistical analysis

For analysis, the final cohort was divided into 2 groups based on age at first diagnosis (<45 years and ≥45 years). Categorical variables were reported as frequencies and percentages. Continuous variables were reported as means and SDs for normally distributed data and medians and interquartile ranges (25th-75th) for variables that were not normally distributed. Normal distribution was assessed using the Shapiro–Wilk test and by visual inspection of histograms. Group differences were measured by the Pearson chi-square or Fisher exact test for categorical variables and the 2-sample *t* test or Wilcoxon rank-sum test for continuous variables. To address that age is inherently continuous, sensitivity analyses were performed modeling age at diagnosis as a continuous variable with baseline BMI, HbA1c, and eGFR using multivariable linear regression. Missing data were handled by data removal.

For each outcome, raw data were plotted over time, with follow-up truncated at 4 years to minimize the influence of sparse observations at later time points (missingness at 4 years was <1%). Generalized estimating equations were fitted using an exchangeable working correlation structure to model longitudinal trajectories. Restricted cubic splines were used to model nonlinear trends in follow-up time, and an interaction between time and age group was included to assess differences in trajectories. All models were adjusted for sex and race. The degrees of freedom for spline terms were selected based on the quasilikelihood under the independence model criterion. β estimates with 95% confidence intervals and *P* values were reported. Predicted values from the best-fitting models were plotted to visualize trajectories by age group. Wald tests were conducted to assess whether the outcome trajectories differed significantly over time between groups. Robust SEs were used to account for within-subject correlation.

We first restricted clustering analyses to young adults with T2D (18-44 years). Candidate baseline variables (age, race/ethnicity, HbA1c, BMI, Cr, eGFR, medications, baseline comorbidities) were first examined using hierarchical clustering (Ward's minimum variance method) in JMP Pro 18 to identify groups of variables with high within-group correlation and clinical relevance. Variables with excessive missingness (>20%) or redundancy (pairwise *r* > 0.80) were excluded. We next applied k-means clustering using Euclidean distance to the standardized variables. The cluster comparison (cubic clustering criterion) and elbow method determined that 3 clusters yielded the best balance of model fit and clinical interpretability. Using these 3 baseline clusters, the frequencies of diabetes-related complications were compared among the 3 clusters in young adults with T2D. The same baseline clustering was then applied to the whole cohort (young and older adults with T2D), with a comparison of diabetes-related frequencies among the 3 clusters. Missingness at random or not was assessed first by visualizing missing data patterns. For each variable with missing data, we created a binary variable for missing vs not missing and then determined whether there were significant differences in the distribution or means of other variables. Results indicated that missingness was not completely at random for several laboratory variables (eg, HbA1c, BMI, GFR at follow-up), which was consistent with clinical care patterns. Statistical significance was assessed at a .05 level, and all tests were 2-tailed. Analyses were conducted using JMP Pro 18.0 and R version 4.4.0 (R Core Team 2024; packages tidyverse, cluster, factoextra, visualization, flexclust, knitr, clustMixType, GGally, tidyverse, gtsummary, readxl, lubridate, mgcv, geepack, splines, and ggeffects).

## Results

### Cohort characteristics

Of the 56 326 included in the final analysis, 23% (n = 13 086) were young adults and 77% (n = 43 240) were older adults (Fig. S1) [15]. Table 1 summarizes the clinical characteristics of the study cohort based on age at first diagnosis. Compared with older adults, young adults with T2D were more likely to report female sex (51% vs 47%, *P* < .001), races other than White (24% vs 16%, *P* < .001), Hispanic ethnicity (21% vs 12%, *P* < .001), and had a higher frequency of obesity at baseline (68% vs 62%, *P* < .001).

**Table 1:** Clinical characteristics and laboratory results at baseline of persons with T2D by age at diagnosis

Clinical variable	Age <45 yrs n = 13 086	Age ≥45 yrs n = 43 240	*P* value	Missing	SMD
Age, years	35 (7)	62 (10)	<.001	0	2.88
Female sex, n (%)	6689 (51)	20 471 (47)	<.001	0	
Race, n (%)			<.001	0	
White	9998 (76)	36 337 (84)			
Black	335 (2)	665 (2)			
Asian	366 (3)	946 (2)			
Pacific Islander	487 (4)	871 (2)			
American Indian	339 (3)	548 (1)			
Other	1561 (12)	3873 (9)			
Hispanic ethnicity, n (%)	2572 (21)	4954 (12)	<.001	3 423	
BMI, kg/m^2^	34 (9)	32 (7)	<.001	0	0.27
BMI categories, n (%)			<.001	0	
Normal (<25 kg/m^2^)	1620 (12)	4759 (11)			
Overweight (25-29 kg/m^2^)	2651 (20)	11 719 (27)			
Obese (≥30 kg/m^2^)	8815 (68)	26 762 (62)			
On treatment for T2D, n (%)	7184 (55)	21 955 (51)	<.001	0	
Oral agent for T2D, n (%)	4580 (35)	15 302 (35)	.414	0	
Insulin, n (%)	4849 (37)	13 848 (32)	<.001	0	
Antihypertensive agent, n (%)	4645 (35)	20 413 (47)	<.001	0	
Family history of T2D, n (%)	751 (6)	1632 (4)	<.001	0	
Follow up, months	31 (0-77)	28 (0-74)	<.001	0	
Laboratory results					
HbA1c, %	7.8 (2.5)	7.3 (2.0)	<.001	0	0.24
C-peptide*^a^*, nmol/L	0.96 (0.66-1.39)	0.86 (0.56-1.29)	.081	55 705	
CKD stage by eGFR, n (%)			<.001	12 535	
1	7383 (75)	10 178 (30)			
2	1692 (17)	13 059 (38)			
3	585 (6)	9334 (28)			
4	103 (1)	947 (3)			
5	124 (1)	386 (1)			
Urine albumin, mg/g, n (%)			<.001	28 353	
<30	4333 (64)	13 964 (66)			
30-300	1777 (26)	5452 (26)			
>300	667 (10)	1780 (8)			

Abbreviations: BMI, body mass index; CKD, chronic kidney disease; eGFR, estimated glomerular filtration rate; HbA1c, hemoglobin A1c; T2D, type 2 diabetes mellitus.

^*a*^Median and interquartile range.

Despite similar baseline C-peptide concentrations, although with substantial missing data, young adults with T2D had higher baseline HbA1c concentrations and were more likely to have insulin prescribed compared with older adults (Table 1). However, young adults with T2D had better baseline renal function despite a slightly higher frequency of severely increased albuminuria (9.8% vs 8.4%, *P* < .001).

### Comorbidities

Young adults with T2D had higher rates of DKA and hypoglycemia and similar frequencies of retinopathy and foot ulcers but were less likely to have hypertension, nephropathy, neuropathy, or PVD compared to older adults (Fig. S2) [15]. The presence of 3 cardiometabolic risk factors (T2D, hypertension, and obesity) occurred in 67% of young adults with T2D compared with 85% of older adults (*P* < .001).

**Figure 1:**
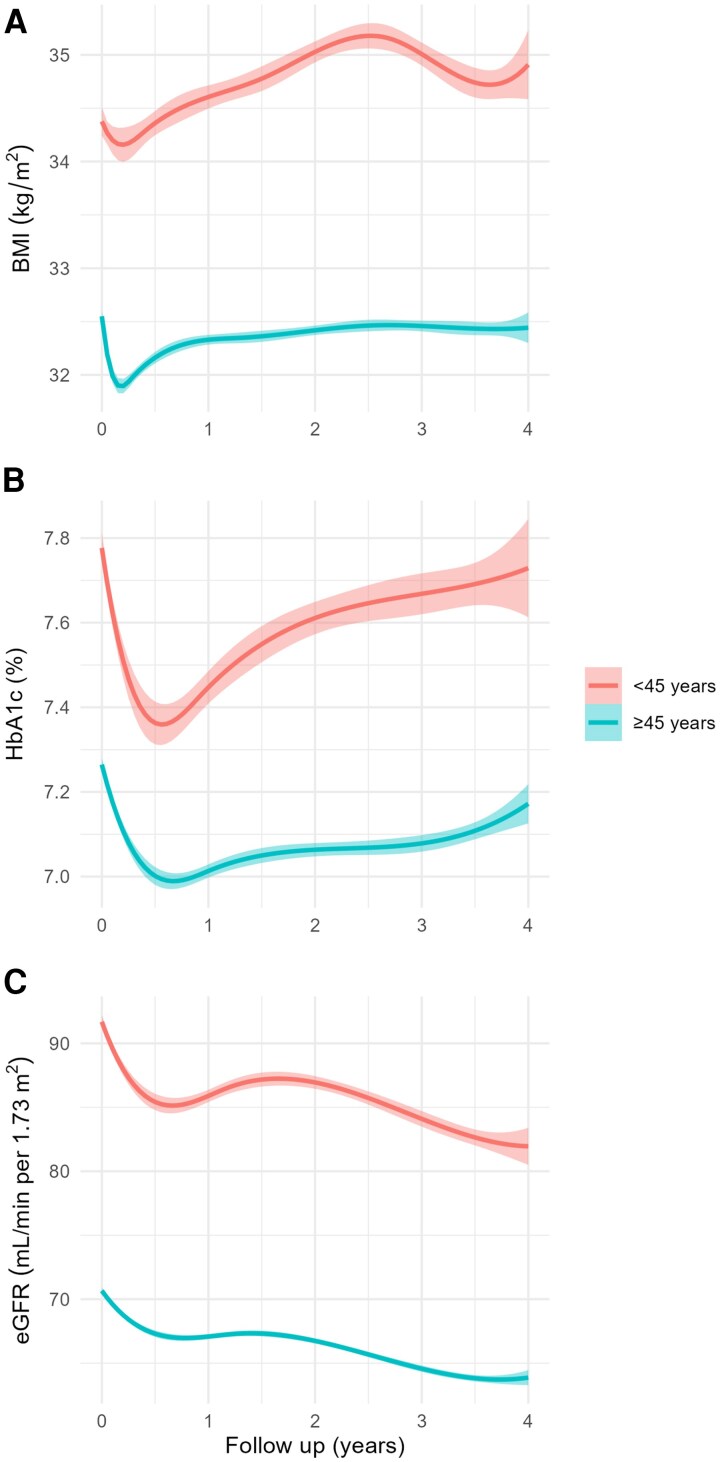
BMI (A), HbA1c (B), and eGFR (C) over a 4-year follow-up interval by age group. Lines indicate the mean, and the shaded area is 1 SD around the mean. *P* < .001 for all time points. Abbreviations: BMI, body mass index; eGFR, estimated glomerular filtration rate; HbA1c, hemoglobin A1c.

Compared with young adults, over time, older adults had significantly lower BMI (*P* < .001), HbA1c (*P* < .001), and eGFR (*P* < .001) [Table 2]. For BMI and HbA1c, the joint Wald test for the interaction between follow-up time and age group was significant (*P* < .001), indicating the trajectories over time differ between age groups, with less pronounced increases in older compared with younger adults (Fig. 1A and 1B). However, the joint Wald test for eGFR was not significant (*P* = .6) (Fig. 1C), signaling a similar decline in eGFR over time. These findings were consistent with our model predictions (Fig. S3) [15]. In sensitivity analyses adjusting for sex, race and ethnicity, modeling age as a continuous variable, younger age was significantly associated with higher baseline BMI (β = −.096, *P* < .001), higher baseline HbA1c (β = −.021, *P* < .001), and higher baseline eGFR (β = −.94 mL/min/1.73m^2^, *P* < .001), consistent with the findings from the categorical age-based analysis. On follow-up, age as a continuous variable was associated with a decrease in BMI (β = −.119, *P* < .001), a decrease in HbA1c (β = −.021, *P* < .001), and a decrease in GFR (β = −.931, *P* < .001) (Fig. S4) [15].

**Table 2:** Results of the model trajectories over time for BMI, HbA1c, and eGFR^a^

Characteristic	β	95% CI	*P* value
BMI			
Age			
<45 years	Reference		
≥45 years	−1.8	−1.9, −1.6	<.001
Sex			
Male	Reference		
Female	1.3	1.2, 1.5	<.001
Race			
White	Reference		
Black	−0.45	−0.91, 0.02	.059
Asian	−5.8	−6.1, −5.5	<.001
Pacific Islander	3.6	3.1, 4.0	<.001
American Indian	−0.10	−0.60, 0.39	.7
Other	−1.5	−1.7, −1.3	<.001
HbA1c			
Age			
<45 years	Reference		
≥45 years	−0.49	−0.53, −0.44	<.001
Sex			
Male	Reference		
Female	−0.39	−0.42, −0.36	<.001
Race			
White	Reference		
Black	0.42	0.30, 0.55	<.001
Asian	0.07	−0.01, 0.15	.087
Pacific Islander	1.1	0.98, 1.2	<.001
American Indian	0.65	0.51, 0.79	<.001
Other	0.31	0.26, 0.36	<.001
eGFR			
Age			
<45 years	Reference		
≥45 years	−23	−23, −22	<.001
Sex			
Male	Reference		
Female	−1.9	−2.3, −1.4	<.001
Race			
White	Reference		
Black	−0.37	−2.2, 1.5	.700
Asian	7.3	5.9, 8.6	<.001
Pacific Islander	−4.9	−6.6, −3.3	<.001
American Indian	2.4	0.09, 4.7	.042
Other	3.8	2.9, 4.6	<.001

Abbreviations: BMI, body mass index; eGFR, estimated glomerular filtration rate; HbA1c, hemoglobin A1c.

^a^Model results do not include spline terms for simplicity.

The proportion of the group defined as at risk for hepatic fibrosis from MASLD by the APRI significantly increased on follow-up in both groups (48% vs 55%, *P* < .01) (Fig. S5) [15]. Unfortunately, the FIB-4 was less reliable in 40% of the cohort due to age ≤35 years or ≥65 years old [18]. Of those who were >35 years old and <65 years old, 16% of young adults with T2D and 36% of those ≥45 years old had a FIB-4 of ≥1.3 (*P* < .01).

### Cluster analysis

**Figure 2:**
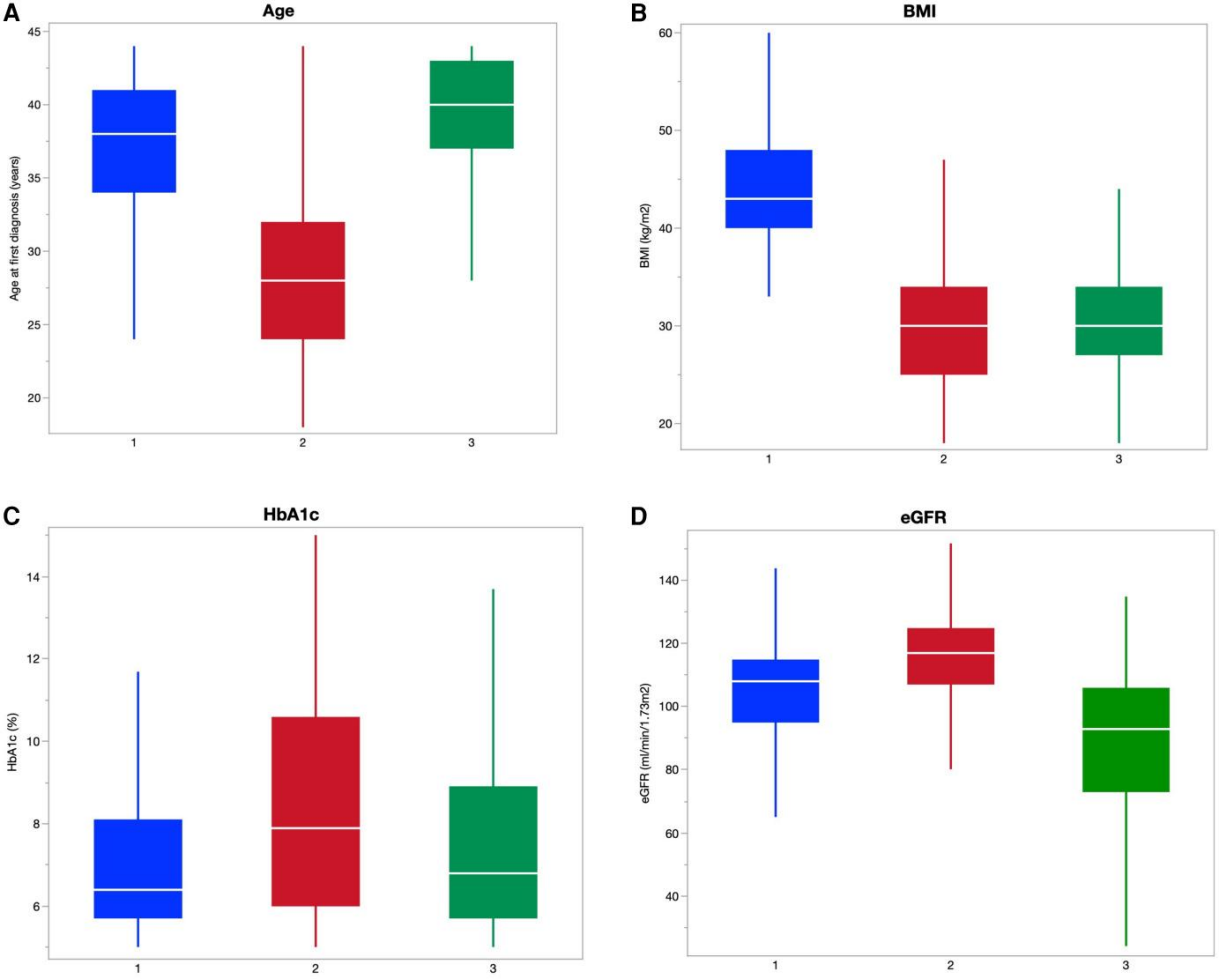
Cluster characteristics in young adults with T2D: distribution of age (A), BMI (B), HbA1c (C), and eGFR (D) for each cluster. BMI, body mass index; eGFR, estimated glomerular filtration rate. *P* < .01 for all comparisons. Abbreviations: BMI, body mass index; eGFR, estimated glomerular filtration rate; HbA1c, hemoglobin A1c; T2D, type 2 diabetes.

After hierarchical analysis of young adults with T2D, baseline clinical variables with the highest effect were age at first diagnosis, baseline BMI, baseline HbA1c, and baseline eGFR. Utilizing these variables, 3 unique baseline phenotypic subgroups in young adults with T2D were created, with results reported in the order of cluster 1, then 2, and then 3 (Fig. 2). Cluster 1 can be described as “obesity predominant” as it had a higher BMI (45 ± 7 vs 30 ± 6 vs 30 ± 5 kg/m^2^, *P* < .01), lower HbA1c (7.2 ± 2.1% vs 8.5 ± 2.8% vs 7.6 ± 2.4%, *P* < .01), and normal eGFR at baseline (Fig. 2 and Table S1) [15]. Cluster 2, or “insulin deficient,” were likely to be the youngest but had a large age range (37 ± 5 vs 28 ± 5 vs 39 ± 4 years, *P* < .01), with mild obesity, significantly elevated HbA1c, and normal eGFR. Cluster 3, or “renal predominant,” was slightly older at first diagnosis but had a significantly lower eGFR at baseline (104 ± 18 vs 115 ± 16 vs 86 ± 27 mL/min/1.73 m^2^, *P* < .01). Cluster 1 represented a lower risk group, with the lowest frequencies of retinopathy, neuropathy, DKA/HHS, hypoglycemia and PVD, and despite a higher BMI on follow-up (43 ± 8 vs 31 ± 7 vs 31 ± 6 kg/m^2^, *P* < .01), the frequency of hepatic fibrosis from MASLD was lower than cluster 3 (50% vs 44% vs 54%, *P* < .01) (Table S1) [15]. Cluster 2 was a younger group with a higher frequency of insulin use (33% vs 49% vs 40%, *P* < .01), highest risk of hypoglycemia (3% vs 8% vs 7%, *P* < .01), and diabetic ketoacidosis (3% vs 14% vs 6%, *P* < .01). On follow up, cluster 3 represented a group with the highest frequency of diabetes-related complications, including nephropathy (43% vs 31% vs 65%, *P* < .01), retinopathy (5% vs 10% vs 15%), and neuropathy (16% vs 15% vs 24%, *P* < .01) (Table S1) [15]. When the baseline phenotypic cluster groups were applied to older adults (Table S2) [15] and the entire cohort (both young and older adults) (Table S3) [15], a similar pattern was noted; however, the “insulin deficient” cluster 2 had higher frequencies of retinopathy and neuropathy and maintained higher frequencies of DKA, HHS, and hypoglycemia (Tables S2 and S3) [15].

## Discussion

Utilizing a large population cohort linked to electronic health records, we demonstrate that T2D in young adults is a major health concern with high frequencies of diabetes-related complications. Young adults with T2D were found in greater numbers in women and racial/ethnic minorities. We also show that compared with older adults with T2D, young adults had worse glycemic control both at diagnosis and at 4 years follow-up, with a higher frequency of obesity, insulin use, hypoglycemia, and DKA/HHS. Age at diagnosis, baseline HbA1c, baseline BMI, and baseline eGFR were clinical risk factors that clustered into 3 unique baseline phenotypic subgroups that were associated with diabetes-related outcomes. A high-risk group was identified, opening an avenue for possible targeted precision therapy early in the disease process.

T2D in young adults is an emerging health concern with rising prevalence rates. In our large cohort of adults with T2D who accessed care at the 2 largest health systems in the state of Utah, 23% were diagnosed before the age of 45 years old. This may be an underrepresentation, as the age at first entry into the database could be later than the actual true age at diagnosis. In fact, altered glucose metabolism can start 10 to 20 years before a diagnosis of T2D is made [19]. Recent data showed a 40% increase in prevalence rates of young-onset T2D in the UK over the past 5 years, with a global rate of 3.8% in 2021 [20]. Like prior reports, we also show that T2D disproportionately affects racial and ethnic minority groups in young adults [2]. However, young adults with T2D and people identifying with racial or ethnic minority groups have been underrepresented in disease-modifying pharmacological studies [21]. This presents a significant hurdle in translating findings into clinical management of T2D and preventing T2D-related comorbidities in a large subset of adults with T2D. The concern is particularly heightened in young adults, where our data highlight worse glycemic control, rising BMI at interval follow-up, and a similar rate of decline in renal function over time. Taken together, these findings suggest a higher burden of diabetes-related complications over the lifetime of a young adult with T2D given an earlier onset of the disease process. With the available evidence, it is not clear whether disease-modifying agents like sodium-glucose co-transporter-2 inhibitors are as effective in young adults compared with older adults with T2D. Safety data have been published in children and very young adults (age 10-24 years) with the use of dapagliflozin [22]. However, there is no data regarding renoprotective effects [22]. Similarly, young adults comprised small subsets in cardiovascular and renal trials of glucagon-like peptide receptor agonists [23, 24]. A recent subgroup analysis of the SURPASS trials (tirzepatide) showed similar treatment effects in young adults as older adults with T2D [25], but no cardiovascular or renal outcome data were reported. In addition, there is a need for pregnancy safety data with these medications, given that T2D affects young women more than young men. A recent population-based cohort analysis suggested comparative safety of sodium-glucose co-transporter-2 inhibitors and glucagon-like peptide receptor agonists with insulin in the periconception period and first trimester of pregnancy [26]. However, the recommendation currently is against the use of these agents in women of reproductive age unless reliable contraceptive measures are used [27].

Young adults with T2D appear to have a more aggressive disease process, with higher rates of comorbidities, faster decline in β-cell function, and lower life expectancy [28]. Despite a younger age, nephropathy, neuropathy and retinopathy were frequent among young adults with T2D in our study. Indeed, significant risk factors associated with increased risk of microvascular complications are prevalent in young adults with T2D likely due worse glycemic control and obesity [10, 29, 30], which further amplifies the decline in β-cell function. β-cell function studies in adolescents with T2D showed lower β-cell reserve and lower insulin sensitivity compared to adults [8, 31]. In epidemiological studies, younger age at diagnosis and higher BMI were associated with a higher rate of deterioration of β-cell function [32, 33]. Utilizing HOMA2-B and fasting C-peptide concentrations in an overweight/obese cohort by Asian BMI thresholds, there was lower β-cell secretion in young adults with T2D (mean age 39.9 ± 7.5 years) compared with older adults with T2D (mean age 60.8 ± 10.6 years) [34]. The lower mean C-peptide concentrations in that study (0.5 nmol/L) compared with ours (mean C-peptide, 0.96 nmol/L) suggest lower endogenous insulin production in their cohort. Despite this, young adults with T2D in our cohort had higher rates of insulin use, hypoglycemia, and DKA/HHS, which are clinically consistent with poorer β-cell function compared with older adults. Further evaluation under controlled settings (eg, fasting vs postprandial) is needed to truly investigate β-cell function in our population.

Renal function decline trajectories during longitudinal follow-up in young adults with T2D can be a harbinger of increased future risks of end-stage kidney disease and cardiovascular morbidity and mortality. While eGFR was always numerically higher in young adults with T2D during follow-up, the rate of decline was similar to that of older adults, suggesting that with an earlier start of eGFR decline, chronic kidney disease will be more prevalent in the future unless preventative measures are used. This finding highlights the need for more aggressive management in young adults with T2D, as the risk of cardiovascular disease and heart failure is increased with chronic kidney disease, regardless of age at diagnosis of T2D [35]. Further, a meta-analysis of observational data with more than 1 million adults showed that a younger age at diagnosis of T2D was associated with an increased risk of death and cardiovascular disease [36]. While our study could not account for cardiovascular disease, overall cardiovascular risk was likely high in both groups in our cohort due to the presence of concomitant cardiometabolic risk factors.

To stratify our cohort, we created 3 baseline phenotypic subgroups with differing trajectories based on clinical variables available to most clinicians (age at diagnosis of T2D, BMI, HbA1c, and eGFR). Prior studies described 5 unique T2D clusters based on antibody status (glutamate decarboxylase antibodies), age at diagnosis, BMI, HbA1c, HOMA2-IR, and HOMA2-B [1, 11]. These novel subgroups have been replicated in other cohorts [37, 38]. However, the parameters used to create these subgroups are not routinely available from recommended clinical assessments. The HOMA2-IR and HOMA2-B calculations require simultaneous fasting insulin, C-peptide, and glucose measurements. Fasting insulin and C-peptide measurements are not routinely measured in the clinic, are not available in rural settings, and are not recommended as routine measures for T2D by the American Diabetes Association Standards of Care [12]. Indeed, in our cohort, too few individuals had these measurements, and we were unable to verify that they were measured fasting. As such, we relied on variables that are available to almost all clinicians. There were some similarities between our clusters and those described previously. Our cluster 1 was similar to the previously described mild obesity–related T2D cluster, and our cluster 2 was similar to the previously described severe insulin-deficient cluster [1, 11]. We describe a “renal predominant” cluster (cluster 3), which is a unique, clinically important group that has a high burden of diabetes-related complications highlighted by a lower eGFR at baseline. With the use of routine clinical variables, these baseline phenotypic clusters are easier to translate into clinical practice and could translate into risk-stratified monitoring—prioritizing weight management with the use of intensive lifestyle intervention and medications (eg, glucagon-like peptide-1 receptor agonists) in cluster 1, early intensification of glycemic therapy in cluster 2, and more intensive renal surveillance with early intervention of renoprotective therapies (eg, angiotensin-converting enzyme inhibitors/angiotensin receptor blockers, sodium–glucose cotransporter 2 inhibitors, and mineralocorticoid agonists) in cluster 3. The use of glucagon-like peptide-1 receptor agonists and sodium–glucose cotransporter 2 inhibitors was limited in our cohort (∼3%), and granular longitudinal treatment data were not available. As such, we were unable to determine whether the observed differences in renal function and comorbidity burden were independent of treatment effects. Accordingly, the identified clusters should be viewed as descriptive baseline phenotypes rather than treatment-adjusted or causal subgroups. In addition, before implementation into clinical practice, prospective validation is required.

Young adults with T2D are at comparable risk for hepatic fibrosis from MASLD as older adults [39]. We therefore also assessed the risk of hepatic fibrosis in young adults with T2D using noninvasive risk stratification tools. Clusters 1 and 3 both had higher frequencies of elevated risk scores for hepatic fibrosis from MASLD. However, our study highlights the limitations of assessing hepatic fibrosis risk in young adults in routine practice. The recommended FIB-4 index to screen for hepatic fibrosis [40, 41] was less reliable due to age thresholds [18] in 40% of our cohort. As such, we used the APRI score, which is not affected by age and is currently the best noninvasive screening tool available to identify hepatic fibrosis in young adults [42]. We show that on follow-up, both younger and older adults had a significant increase in APRI scores, with roughly half of both younger (48%) and older adults (55%) exhibiting an elevated APRI score. In contrast, the FIB-4 showed that only 16% of young adults were at a higher risk for fibrosis on follow-up (FIB-4  ≥  1.3); however, 46% of young adults were <35 years old and were excluded from this estimation. Thus, there is a need to develop better methods to identify young adults with T2D at highest risk for hepatic fibrosis using routine clinical variables because the currently recommended method (ie, FIB-4) is likely to underestimate their risk. The use of our clusters may serve as 1 step closer to improving the identification of young adults at higher risk of hepatic fibrosis. However, confirmation of hepatic fibrosis with liver imaging (eg, vibration-controlled transient elastography or magnetic resonance elastography) is needed for accurate diagnosis.

Our study has several strengths, including a large database with longitudinal data that includes a significant proportion of Latinx individuals and identification of T2D through a novel, deeply phenotyped database (UDDb) [14] with a large proportion of young adults with T2D. However, database-driven studies are based on the identification of diagnoses by diagnosis codes, which present several limitations, including residual confounding. As such, observed differences represent associations with real-world differences rather than causal effects. In particular, the type of diabetes was not always confirmed by laboratory parameters (autoantibody testing and C-peptide concentrations). The UDDb was also curated based on the ratio of diagnosis codes. The exact ratio of these codes for each individual is not readily accessible, limiting sensitivity analyses based on diagnosis code assignment. Age at first diagnosis was presumed to be the age at first entry into the database, given that the database captures 85% of the population of Utah [14]. However, the age at first diagnosis can be lower than was captured in the database; therefore, the true duration of diabetes was unknown. The age at which young adults are defined is also subject to debate, with studies using varying thresholds [1-3]. The age of <45 years was chosen based on prior data, including the novel subgroups in young-onset T2D [1, 43, 44]. The African American population in Utah is low; thus, the cohort does not reflect the diversity seen across the United States. Standardized follow-up data at regular intervals were not available for most individuals, as this was dependent on clinical care patterns (Table S5) [15]. As such, the rate of transitions of complications and many objective outcomes cannot be reliably calculated. Glycemic control was assessed by the HbA1c over time, which is influenced by a multitude of factors, including biologic disease, healthcare access, medication use, lifestyle, and provider-level practices. The absence of detailed data on lifestyle management, socioeconomic determinants, education, medication adherence, lipid parameters, and β-cell function data limits the ability to fully account for these factors and contribute to residual confounding. Given the lack of β-cell function data, these clusters cannot replace the already established 5 clusters previously described [11] or determine etiologic pathways. Finally, independent replication and validation of the clusters in another cohort are needed, with more in-depth prospective follow-up to determine clinical significance and applicability to individualized patient care.

In conclusion, T2D among young people disproportionately affects women and racial/ethnic minorities. Young adults also have a high risk of comorbidities, including chronic kidney disease. Baseline lower eGFR suggests a higher risk group with high frequencies of multiple diabetes-related complications, including nephropathy, retinopathy, and neuropathy. Further research is needed to develop and implement precision prognostics and precision therapy to prevent diabetes-related comorbidities in adults with T2D, in particular, young adults with T2D.

## Data Availability

The data that support the findings of this study are available from the authors, but restrictions apply to the availability of these data, which were used under license from the University of Utah and the Utah Population Database for the current study and so are not publicly available. Some or all datasets generated during and/or analyzed during the current study are not publicly available but are available from the corresponding author on reasonable request and with permission from the University of Utah and the Utah Population Database.
